# Use of Chinese Medicine Reduces the Development of Cervical Cancer from Pap Smear-Diagnosed Cervical Dysplasia: A Case-Control Study

**DOI:** 10.1155/2017/4082630

**Published:** 2017-12-31

**Authors:** Li-Ling Shen, Chih-Hsin Muo, Shan-Yu Su, Donald E. Morisky

**Affiliations:** ^1^Department of Chinese Medicine, China Medical University Hospital, Taichung 40447, Taiwan; ^2^Department of Public Health, China Medical University, Taichung 40402, Taiwan; ^3^Management Office for Health Data, China Medical University Hospital, Taichung 40402, Taiwan; ^4^School of Post-Baccalaureate Chinese Medicine, College of Chinese Medicine, China Medical University, Taichung 40402, Taiwan; ^5^Department of Community Health Sciences, UCLA Fielding School of Public Health, Los Angeles, CA 90095-1772, USA

## Abstract

The Pap test diagnosed cervical dysplasia, which could recover to normal or progress to cervical cancer (CC), is an early stage of cell abnormality before CC. This case-control study analyzed the differences in the risk to develop CC between Chinese medicine (CM) users and nonusers among women who had ever been diagnosed as having cervical dysplasia. A total of 750 CC patients with a cervical dysplasia history were collected between 1998 and 2011 from National Health Insurance Research Database, and controls were women with cervical dysplasia history but did not develop CC. Adjusted odds ratio (aOR) for developing CC was assessed using multivariable logistic regression after adjusting for age, urbanization of residence, and occupation. The proportion of using CM among CC patients was lower than that among CC nonpatients, with an aOR of 0.8. By analyzing the relationship between CC development and the frequency of CM usage, the trend test revealed a significant decreasing trend for developing CC among high-frequency CM users. Moreover, the most frequently used single herb high-frequency was* Rheum palmatum *(Da-Huang). The usage of CM might be an effective complementary method to prevent uterine cervix from progressing to CC after cervical dysplasia has occurred.

## 1. Introduction

Cervical cancer (CC) is a prevailing cancer in women, with about 528,000 annual cases and 266,000 deaths per year worldwide [1]. The incidence and the mortality rate depend on the penetration of CC screening programs, which is the most important strategy to prevent CC [2]. In Taiwan, the government launched a nationwide cervical screening program in July 1995, and since then, annual Pap smear screening test has been freely provided to women over 30 years old [3]. By detecting the very early stage abnormal cell changes in the uterine cervix, which is called “cervical dysplasia,” Pap smear test reduces the development of invasive CC years later because of the close follow-up and early management of the dysplasia [4].

The Pap test diagnosed early stage dysplastic cervix, including atypical squamous cells of undetermined significance, low-grade squamous intraepithelial lesion, atypical squamous cells, and high-grade squamous intraepithelial lesion, could either recover to normal or progress to CC. Reported rate of spontaneous regression among cervical dysplasia ranges from 33% to 60%, and the rate of progression to invasive CC ranges from 1% to 12%. Therefore, instead of treatment, close follow-up with repeated Pap tests every three months and colposcopy biopsy are usually suggested for cervical dysplasia [5]. When the cervical dysplasia keeps appearing for 12 months or progresses to the next stage, laser treatment or endocervical curettage combined with conization, are indicated [6]. After local excision, close follow-up is still needed because there is still recurrent dysplasia that has been reported in 0.3% to 23% of women with clear excisional margins [7, 8].

Chinese medicine (CM) is commonly used as a complementary medicine in the treatment of cancer and has been reported to reduce side effects and reinforce the efficacy of conventional treatment [9, 10], as well as increase survival rate of cancer patients [11]. Regarding preventing cancer, large number of natural products and molecules extracted from herbs have been shown in laboratories to be potential to prevent cancer [12]. Theses natural products were reported to exert the anticancer activity via inhibiting cancer cell proliferation, arresting cell cycle, inducing apoptosis, inhibiting epithelial-mesenchymal transition, regulating immune function [13], and exerting antioxidative and anti-inflammatory effects [14] in cell and animal studies. However, epidemiological literature regarding whether CM prevents cancer from happening is limited. The only large case number study conducted on the prevention of cancer by CM found that CM might prevent tamoxifen-caused endometrial cancer in breast cancer patients [15]. There is still no concrete evidence in the literature about whether CM prevents the development of CC from early stage of cervical dysplasia.

As the most important complementary medicine in Taiwan, since 1995 CM have been covered by the National Health Insurance (NHI), which is a health insurance system established by the government [16]. The database released by NHI contains nationwide medical reimbursement information, which provides researchers to follow patients for several years to study patterns, treatments, and prognosis of diseases [17]. In order to investigate the relationship between the development of CC from cervical dysplasia and the use of CM, the present population-based case-control study extracted women who had ever been diagnosed as cervical dysplasia by Pap test from the NHI database. Then, the differences in CM usage between cervical dysplasia patients who consequently developed CC and who did not developed CC were analyzed.

## 2. Materials and Methods

### 2.1. Study Design and Study Population

**Figure 1:**
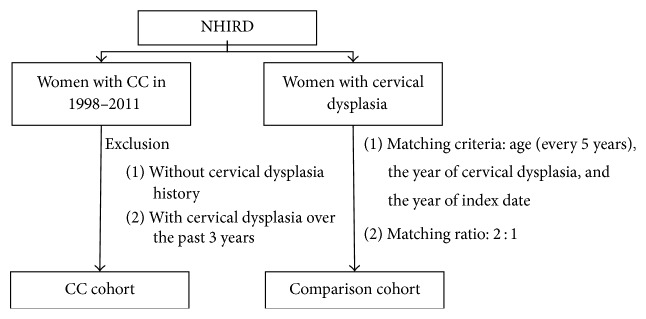
Flow chart for abstracting study subjects. NHIRD, National Health Insurance Research Database, and CC, cervical cancer.

This case-control study used the National Health Insurance Research Database (NHIRD) released by the Bureau of National Health Insurance. Almost all Taiwanese population joins this NHI program, with a coverage ratio of over 99%. NHIRD contained outpatient and inpatient medical claims of each insurant from 1997 to 2011. By reason of the Personal Information Protection Act, the NHIRD was a second-hand database with identity number deidentified. Diseases were defined according to the International Classification of Diseases, Ninth Revision, Clinical Modification (ICD-9-CM) in NHIRD. We collected CC patients (ICD-9-CM 180) with a cervical dysplasia history (ICD-9-CM 622.1) from 1998 to 2011. Patients with CC development within three years after the cervical dysplasia diagnosis were excluded. The index date was defined as the date of CC diagnosis. Approximately twofold randomly selected controls from women with cervical dysplasia but without CC development were frequency-matched with CC patients on age stratum (every 5 years, e.g., 20–24, 25–29, and 30–34), the year of cervical dysplasia diagnosis, and the year of index date (Figure 1). Patients with CM treatment for more than or equal to 14 days between the cervical dysplasia date and the index date were defined as CM users. The demographic confounding variables included age (20–39, 40–49, 50–59, and more than 60 years), urbanization of residence (urban and rural), and occupation (homemaker, white collar, and blue collar).

### 2.2. Statistical Analysis

All analyses were performed using SAS software version 9.4 (SAS Institute, Cary, NC). A two-tailed *p* value of less than 0.05 was defined to be statistical significant.* Chi*-square test was used to examine the differences in categorical variables (including age subgroup, urbanization, and occupation) between CC patients and controls, and the *t*-test was used to examine the differences in continuous variables. Odds ratio (OR) and 95% confidence intervals (CIs) of CC were assessed using multivariable logistic regression model after adjusting for age, urbanization, and occupation. The association between the development of CC and the frequency of CM usage was estimated by dividing CM users into high-frequency group and low-frequency group. Patients received CM for more than 30 days per year were defined as high-frequency users and the others were defined as low-frequency users. The trend test and multivariable logistic regression model were used to test the association between the frequency of CM usage and CC development.

## 3. Results

### 3.1. Characteristics of Study Subjects

A total of 750 CC patients and 1498 CC nonpatients who had been diagnosed as cervical dysplasia were extracted from the NHIRD, with the mean age of 56.2 ± 13.2 years (Table 1). There were no differences in age and urbanization of residence between CC patients and nonpatients. On the other hand, the proportion of white collar workers among CC patients was less than that among CC nonpatients (28.0% versus 37.5%), but the proportions of blue collar workers (41.9% versus 37.4%) and homemakers (30.1% versus 25.1%) among CC patients were higher than CC nonpatients.

### 3.2. CM Usage between CC Patients and CC Nonpatients

Compared to CC nonpatients, there was a lower proportion of CM users among CC patients (19.9% versus 24.0%), with an aOR of 0.80 (95% CI = 0.64–0.99) after controlling for age, urbanization of residence, and occupation (Table 2). Stratification analysis showed that the biggest difference in CM usage happened in the stratum of patients aged 50–59 years (aOR = 0.66, 95% CI = 0.44–0.99). There were no differences in using CM between CC patients and nonpatients in subgroups of urbanization of residence and occupation.

### 3.3. Frequency of CM Usage and the Development of CC

Among CC patients, 80.1% of them were CM nonusers, and others were CM users, composed of 10.1% of high-frequency CM users and 9.73% of low-frequency CM users. On the other hand, among CC nonpatients, 76% were CM nonusers, and others were CM users, composed of 13% of high-frequency CM users and 11.0% of low-frequency CM users (Table 3). Furthermore, the risk of developing CC decreased with the increase of CM using frequency. CC patients had an aOR of 0.86 (95% CI = 0.64–1.16) for being low-frequency CM users and had an aOR of 0.74 (95% CI = 0.56–0.98) for being high-frequency users (trend test *p* = 0.03), compared to CC nonpatients (Table 3).

### 3.4. Prescription Patterns of CM among High-Frequency CM Users

The top ten most frequently prescribed CM singles and formulas for CM high-frequency users are listed in Table 4. The most frequently prescribed CM single was* Rheum palmatum *(Da-Huang), dominating 2.20% of the total CM single prescription, with a mean daily dose of 0.51 g and a mean yearly prescription of 67.6 days. The second commonly prescribed herb was* Salvia miltiorrhiza *(Dan-Shen), dominating 1.96% of total CM single prescriptions, with a mean dose of 1.1 g per day and 43.98 days per year. The third most frequently prescribed single was* Corydalis yanhusuo *(Yan-Hu-Suo), dominating 1.89% of the total CM single prescriptions, with a mean dose of 1.12 g per day and 34.6 days per year. The most commonly prescribed formula was Jia-wei-xiao-yao-san, occupying 4.03% of the total CM formula prescriptions, with a mean dosage of 4.21 g per day and 67.64 days per year. The second frequently prescribed formula was Chuan-qiong-cha-tiao-san, dominating 2.50% of the total CM formula prescriptions, with an average dose of 3.92 g per day and 44.72 days per year. The third frequently prescribed formula was Ge-gen-tang, accounting for 1.74% of the total CM formula prescriptions, with a mean daily dose about 4.12 g per day and 32.35 days per year.

## 4. Discussion

It is well known that herbal medicine, including CM, plays an important role in the treatment of cancer. Herbs used in CM have been shown to help in the immune function enhancement and survival rate improvement in cancer patients [18, 19]. In order to investigate CM effects on the development of cancer, this case-control study examined the relationship between CM utilization and the CC development from cervical dysplasia. The results revealed that, after cervical dysplasia has occurred, those who subsequently developed CC used less CM than those who did not develop CC. Moreover, the longer the patients took CM, the less the possibility was for CC development from cervical dysplasia.

Among cervical dysplasia patients, there were no differences in age and urbanization of residence between who developed CC later and who did not develop CC. Otherwise, there were less proportions of white collar workers, while there were more proportions of blue collar workers and homemakers in patients who developed CC. A higher CC screening participation has been reported among women with qualified occupation than among women who never worked [20]. This implies a low compliance to medical follow-up among homemakers and might explain the high CC development among homemakers found in this study.

The logistic regression model revealed that, after being diagnosed with cervical dysplasia, patients who consequently developed CC used less CM than who did not develop CC, with an aOR of 0.8. In most of the stratified subgroups, the aORs for using CM in CC patients ranged from 0.71 to 0.81 compared to CC nonpatients. However, there was no statistical significance in using CM between CC patients and nonpatients in almost all subgroups except in the subgroup aged between 50 and 59. We speculated that the inability to reveal statistical significance was due to the small case number of each subgroup after stratification. On the whole CC patients were less likely to use CM than CC nonpatients after cervical dysplasia had been diagnosed. The result implied that CM might play a role in the prevention of CC from cervical dysplasia. Moreover, the trend test showed that CC patients had a smaller aOR for using CM high-frequently than for using CM low-frequently, implying that the prevention effect might be dose-dependent. As for preventing cancer by CM, a previous population-based study reported that CM might prevent tamoxifen-caused endometrial cancer in breast cancer patients. In that study, among breast cancer patients who take tamoxifen, CM users were less likely to develop endometrial cancer than CM nonusers, with a hazard ratio of 0.61. [15]. Since there are no other large case number studies regarding preventing cancers by CM, more future works on other cancers will be needed.

Among the top ten CM single herbs used by the high-frequency CM users,* Rheum palmatum *(Da-Huang) was the most highly used one.* Rheum palmatum *(Da-Huang), which is used to clear heat and resolve toxin by TCM practitioners, has been shown to possess anti-inflammatory and anticancer ability [21]. Moreover, a molecular extracted from* Rheum palmatum *(Da-Huang), emodin, was found to inhibit the growth of CC cells by inducing apoptosis through the intrinsic mitochondrial and extrinsic death receptor pathway [22]. The second frequently prescribed single herb was* Salvia miltiorrhiza *(Dan-Shen), which is used to improve the stasis status, a common condition existing in cancer pathogenesis according the theory of traditional CM [23, 24].* Salvia miltiorrhiza *(Dan-Shen) has also been found to possess anti-inflammatory and anticancer activities in several cancer cell lines, not only via inducing apoptosis of cancer cell but also via inhibiting the estrogen receptor signal pathway, indicating it might influence the stimulation of estrogen on CC cells [25]. The third frequently prescribed single was* Corydalis yanhusuo* (Yan-Hu-Suo), which is also used traditionally to remove stasis and has been found to be with an antiproliferative effect in cancer cells through the combined actions of its two main compounds, named tetrahydropalmatine and berberine [26]. The fourth frequently prescribed single herb was* Scutellaria baicalensis *(Huang-Qin), from which baicalein is isolated and has been reported to have the function of suspending the cell cycle and inhibiting the proliferation of SiHa and HeLa cervical cancer cells [27]. The fifth frequently used CM single was* Zizyphi spinosi semen* (Suan-Zao-Ren), which is widely used for the treatment of insomnia. Its active compound, Sanjoinine A, has been shown to increase sleep rate and sleep time [28]. It has been reported that sleep disturbance is one of the risk factors for cancers [29]; therefore,* Zizyphi spinosi semen* (Suan-Zao-Ren) might help in the prevention of cancer through its sedative effect.

The top one CM formula used by CM users in the present study was Jia-wei-xiao-yao-san, which has been reported to improve the survival in a variety of cancer patients [30]. Moreover, Jia-wei-xiao-yao-san is commonly used as a substitute for hormone replacement therapy to treat climacteric syndrome, including symptoms of depression, insomnia, and hot flushes [31, 32]. The second commonly used CM formula was Chuan-qiong-cha-tiao-san, of which the main herb is* Ligusticum chuanxiong *(Chuan-qiong). With its antioxidative function,* Ligusticum chuanxiong *(Chuan-qiong) possesses anticancer effects through the induction of apoptosis and inhibition of DNA damage [33]. The third frequently used CM formula was Ge-gen-tang, whose major herb is* Puerariae radix* (Ge-gen). The main component of* Puerariae radix* (Ge-gen), puerarin, has been reported to decrease the activities of tumor by inhibiting tumor growth [34] and inducing apoptosis [35]. The fourth and fifth commonly prescribed CM formulas were Suan-zao-ren-tang and Tian-wang-bu-xin-dan. Both of the formulas are used for the treatment of sleep disorder in traditional CM, and both contain the herb of* Zizyphi spinosi semen* (Suan-Zao-Ren), which was the fifth frequently prescribed single herb in this study. Therefore, Suan-zao-ren-tang and Tian-wang-bu-xin-dan might prevent cancer via a similar mechanism to* Zizyphi spinosi semen* (Suan-Zao-Ren). By analyzing the top five CM singles and formulas used by high-frequency CM users, we hypothesized that besides herbs that are pharmacologically proved to possess anticancer activities, herbs that are used to treat mental symptoms are also important in the prevention of cervical cancer. This hypothesis could be supported by the theory that unstable mental status could cause immune dysfunction [36]. Moreover, in menopausal women those herbs that treat menopausal symptoms and stabilize mental status might decrease the use of estrogen, which is one of the risk factors for CC [37]. We speculated that this could also be a reason that CM users were more unlikely to develop CC compared to CM nonusers, especially among women aged from 55 to 59 years.

The basic limitation of the present study was the retrospective design, which could only reveal a correlative association between CC and CM usage, but it cannot show a causal relationship between these two variables. The second limitation is that the NHI database is a collection of insurance reimbursement data, instead of being designed for research. Therefore, there might be incorrect data and missing data in the database. Third, the database only records medical prescription, instead of real medicine taking, so there may be biases in counting dosage. Fourth, since the national insurance system did not pay other forms of complementary medicine, such as Ayurveda, hypnosis, aroma, and homeopathy, we could not adjust for variables of other types of complementary medicine. And fifth, although models in this study have been adjusted for age, urbanization, and occupation, these confounding factors might still affect the efficacy of CM. Besides, confounding factors mentioned in the second, third, and fourth limitations could not been put into model for adjusting; therefore, there might be bias in this study. On the other hand, the advantage of this study design is that a large number of subjects containing the whole population were recruited at the same time. The present study provided evidence for the fact that the use of CM negatively correlated with CC development and found possible CM candidates which might be beneficial to cervical dysplasia patients.

In conclusion, this case-control retrospective study showed that, after the dysplasia had been diagnosed, patients might have less opportunity to progress to CC if they take CM. Moreover, high-frequency CM users gained even lower risk of CC than low-frequency users. This study suggested that the usage of CM might be a complementary method to prevent the development of CC from cervical dysplasia.

## Figures and Tables

**Table 1:** Demographic factors of cervical cancer patients and nonpatients who had ever been diagnosed as cervical dysplasia.

	CC patients *N* = 750		CC nonpatients *N* = 1498		*p* value^*∗*^
	*n*	%	*n*	%	
Age, year					0.99
20–39	72	9.60	144	9.61	
40–49	202	26.9	404	27.0	
50–59	216	28.8	432	28.8	
>59	260	34.7	518	34.6	
Mean (SD)	56.2 (13.2)		56.0 (13.2)		0.53
Urbanization					0.07
Urban	424	56.5	906	60.5	
Rural	326	43.5	592	39.5	
Occupation					<0.0001
Homemaker	226	30.1	376	25.1	
White collar	210	28.0	562	37.5	
Blue collar	314	41.9	560	37.4	

^*∗*^ *Chi*-square test and *t*-test. CC, cervical cancer.

**Table 2:** Odds ratio for using CM in cervical cancer patients and nonpatients stratified by demographic subgroups.

	CC patients		CC nonpatients		
	*n*	%	*n*	%	aOR (95% CI)
CM usage	149	19.9	359	24.0	0.80 (0.64–0.99)^*∗*^
Age, year					
20–39	19	26.4	38	26.4	0.98 (0.51–1.89)
40–49	43	21.3	112	27.7	0.71 (0.47–1.06)
50–59	40	18.5	111	25.7	0.66 (0.44–0.99)^*∗*^
>60	47	18.0	98	18.9	0.95 (0.65–1.40)
Urbanization					
Urban	88	20.8	226	24.9	0.81 (0.61–1.07)
Rural	61	18.7	133	22.5	0.76 (0.54–1.08)
Occupation					
Homemaker	41	18.1	86	22.9	0.73 (0.48–1.11)
White collar	43	20.5	152	27.1	0.71 (0.48–1.04)
Blue collar	65	20.7	121	21.6	0.92 (0.65–1.29)

Adjusted for age, urbanization, and occupation. CC, cervical cancer; CM, Chinese medicine. ^*∗*^*p* < 0.05 in the logistic regression model.

**Table 3:** Adjusted odds ratio for using CM in cervical cancer patients and nonpatients among CM nonusers, low-frequency CM users, and high-frequency CM users.

	CC patients		CC nonpatients		aOR (95% CI)
	*n*	%	*n*	%	
CM nonuser	601	80.1	1139	76.0	1.00
CM users					
Low frequency	73	9.73	164	11.0	0.86 (0.64–1.16)
High frequency	76	10.1	195	13.0	0.74 (0.56–0.98)^*∗*^
*p* for trend					0.03

Adjusted for age, urbanization, and occupation. ^*∗*^*p* < 0.05 in the logistic regression model.

**Table 4:** Top ten CM singles and formulas prescribed for high-frequency CM users.

	%	Days/year	Daily dose (g)
Single			
*Rheum palmatum *(Da-Huang)	2.20	67.60	0.51
*Salvia miltiorrhiza* (Dan-Shen)	1.96	43.98	1.10
*Corydalis yanhusuo* (Yan-Hu-Suo)	1.89	34.60	1.12
*Scutellaria baicalensis* (Huang-Qin)	1.88	36.91	0.99
*Zizyphi spinosi semen* (Suan-Zao-Ren)	1.70	45.20	1.39
*Cyperus rotundus* (Xiang-Fu)	1.50	44.11	0.96
*Fritillaria taipaiensis* (Bei-Mu)	1.47	33.81	1.07
*Angelica dahurica* (Bai-Zhi)	1.33	30.91	1.10
*Platycodon grandiflorus* (Jie-Geng)	1.21	26.48	0.95
*Eucommia ulmoides* (Du-Zhong)	1.19	32.33	1.14

Formula			
Jia-wei-xiao-yao-san	4.03	67.64	4.21
Chuan-qiong-cha-tiao-san	2.50	44.72	3.92
Ge-gen-tang	1.74	32.35	4.12
Suan-zao-ren-tang	1.67	41.28	3.77
Tian-wang-bu-xin-dan	1.55	43.56	3.67
Du-huo-ji-sheng-tang	1.54	40.27	4.66
Zhi-gan-cao-tang	1.53	42.96	3.51
Shu-jing-huo-xie-tang	1.50	34.45	4.59
Xiang-sha-liu-jun-zi-tang	1.50	43.48	4.02
Ban-xia-xie-xin-tang	1.48	32.41	3.69

## Abbreviations

aOR: Adjusted odds ratio
CC: Cervical cancer
CIN: Cervical intraepithelial neoplasia
CM: Chinese medicine
NHI: National Health Insurance
NHIRD: National Health Insurance Research Database.
